# High Prevalence of Skin Diseases and Need for Treatment in a Middle-Aged Population. A Northern Finland Birth Cohort 1966 Study

**DOI:** 10.1371/journal.pone.0099533

**Published:** 2014-06-09

**Authors:** Suvi-Päivikki Sinikumpu, Laura Huilaja, Jari Jokelainen, Markku Koiranen, Juha Auvinen, Päivi M. Hägg, Erika Wikström, Markku Timonen, Kaisa Tasanen

**Affiliations:** 1 Department of Dermatology, Medical Research Center, University of Oulu and Oulu University Hospital, Oulu, Finland; 2 Institute of Health Sciences, Faculty of Medicine University of Oulu, Oulu, Finland; 3 Unit of General Practice, Oulu University Hospital, Oulu, Finland; Oslo University Hospital, Ullevål, Norway

## Abstract

To determine the overall prevalence of skin diseases a whole-body skin examination was performed for 1,932 members (46-years of age) of the Northern Finland Birth Cohort (NFBC 1966), which is a comprehensive longitudinal research program (N = 12,058). A high prevalence of all skin diseases needing treatment was found (N = 1,158). Half of the cases of skin findings were evaluated to be serious enough to require diagnostic evaluation, treatment or follow-up either in a general health care, occupational health care or a secondary care setting. The remaining half were thought to be slight and self-treatment was advised. Males (70%) had more skin diseases needing treatment than females (52%) (P<0.001). The most common skin finding was a benign skin tumor, which was found in every cohort member. Skin infections (44%), eczemas (27%) and sebaceous gland diseases (27%) were the most common skin diseases in the cohort. Moreover, skin infections and eczemas were more commonly seen in the group with low education compared to those with high education (P<0.005). The results strengthen the postulate that skin diseases are common in an adult population.

## Introduction

Skin diseases are common and they are found in more than half of adult population [1]. The most common skin diagnoses are benign tumors, eczemas, viral warts, sebaceous gland diseases and fungal infections [1], [2], [3]. About one in every three of all patients at general practitioner suffers from a skin disorder [2], [4]. Most dermatologic diseases are chronic in nature and decrease the daily quality of life [2], [5].

Despite the high morbidity and substantial load for primary health care, epidemiological studies addressing the overall prevalence of skin diseases at population level (among a healthy population) are sparse and controversial [1], [3], [6], [7]. In a pioneering study by Rea and co-workers over one-fifth out of 614 study cases had a moderate or severe skin lesion, and age, sex, and social class trends in prevalence were found in certain groups of skin disease [1]. There was a slight (61% versus 48%) female predominance in the prevalence of skin diseases [1]. Another large epidemiologic study by Johnson and Roberts was also conducted as early as in the 1970s [1], [3]. In this diverse Health and Nutrition Examination Survey nearly one-third of persons 1–74 years of age had at least one skin condition that required an evaluation by a physician and the prevalence of skin diseases increased rapidly with age [3]. In a recent German study the prevalence of skin diseases and the need of treatment were investigated by means of a wide cross-sectional analysis of 90,880 employed study cases [6]. Need of treatment was higher in males (31% versus 22%) compared with females [6]. Another recent analysis of 1457 randomly selected adults in Australia revealed following prevalences of skin diseases: eczemas 31.6%, psoriasis 6.6% and acne 12.8%, but the association between skin diseases and socioeconomic classes was not addressed [7].

Due to the scarce epidemiological-level evidence of the prevalences of skin diseases we decided to study the overall prevalence of all skin disorders among an unselected middle-aged population in a developed country, which was the primary aim of the study. Furthermore, the association between skin disorders and sex or socioeconomic status is poorly understood. Consequently, another aim was to investigate the possible sex differences in skin diseases and finally, the association between skin diseases and socioeconomic status.

## Materials and Methods

### Study population

We performed a comprehensive birth-cohort study to find out cross-sectional point prevalence of skin diseases in a middle-aged population. The study population is part of the Northern Finland Birth Cohort 1966 study (NFBC 1966), which is a longitudinal research program in the two northernmost provinces in Finland. NFBC 1966 included initially all 12,058 children whose expected time of delivery was in the year 1966. The whole NFBC 1966 cohort has been evaluated regularly since birth by means of health questionnaires and clinical examinations.

In connection with a 46-year follow-up survey, altogether 3,181 persons currently living in a given geographical area (in the city of Oulu and within 100 km of it, including rural areas) were asked to the clinical examination; 1,932 of them were reached (60.7%) to form the skin study population, which is here called the cohort. Cohort members were 45–47 years of age at this stage.

### Ethics Statement

The Ethical Committee of the Northern Ostrobothnia Hospital District approved the study (§94/2011), which was performed according to the Helsinki Declaration of 1983. Written consent for scientific purposes was received from all participants.

### Clinical examination

Between April 2012 and May 2013, all 1,932 study cases were evaluated by multidisciplinary examination including body index, blood pressure, heart echo recording, dental status and several other health measurements. For the purpose of this study, full dermatologic status was comprehensively determined at an out-hospital visit. This examination began with visual observation of the whole skin and was performed by a specialist in dermatology or an experienced resident. Skin tumors were further investigated by a dermatoscope. Bacterial, viral and fungal skin infections were diagnosed on the basis of clinical picture without laboratory investigations. This group of diseases includes virus warts, folliculitis, onychomycosis and tinea pedis. Inflammatory skin diseases like atopic eczema were classified by an international eczema area and severity index (EASI-index, 0–72 points) consisting of assessment of redness, thickness, scratching and lichenification of eczema in four different body region, as was psoriasis using a psoriasis area and severity index (PASI-index, 0–72 points) which combines the severity and percentage of the affected skin area [8], [9]. International classification of disease characteristics was used for rosacea, acne vulgaris and androgenetic alopecia [10], [11], [12], [13]. Location and duration of all skin symptoms and their severity were recognized.

### Registering of findings and the definition of need of treatment

All cutaneous findings were documented in a computerized database. For additional analysis, the cohort members were classified into four subgroups according to their need of further interventions: I) Subjects with no further care needed, II) those who would recover with self-treatment, III) those to whom a visit at general practitioner was recommended and IV) cases that required further treatment by a dermatologist. This classification was based on a clinical whole body examination. It was decided for every study case whether any follow-up or treatment was required; the chosen treatment was based on cutaneous findings and an expert opinion concerning the accepted clinical practice in dermatology. The group *“no further care needed”* included for example benign tumors, androgenetic alopecia and erytematoteleangiectatic rosacea. The study cases suspected of presenting any skin malignancy or premalignant finding were referred to the Department of Dermatology, University Hospital of Oulu, for biopsy or other further examination and treatment. All other skin diseases requiring treatment, diagnostic investigations or follow-up were referred to primary health care units. The study cases with multiple pigmented nevi were advised to follow their nevi and to protect their skin against sun radiation.

### The classification of education level

The association of the socioeconomic status with the prevalence of skin diseases was investigated by analyzing the relationship between the level of education and the cutaneous findings and diseases. The cohort members were classified in three subgroups of education: basic, secondary and tertiary education (Table 1). The information about education level was obtained from National Education Register [14] and was supplemented with self-reported questionnaires.

**Table 1:** Characteristics of the study population and the classification of need of treatment according to found skin disease.

	Total	Male	Female	
	N = 1930	N = 894 (46.3%)	N = 1036 (53.7%)	
	n (%)	n (%)	n (%)	*P*-value*
*Marital status*				0.036
Married/cohabitant	1465 (78.3)	689 (79.7)	776 (77.1)	
Single	396 (21.2)	175 (20.2)	221 (22.0)	
Widower/widow	10 (0.5)	1 (0.1)	9 (0.9)	
*Educational level* #				0.149
Basic (9 years)	54 (2.8)	30 (3.4)	24 (2.3)	
Secondary (10–12 years)	1130 (58.6)	535 (59.8)	595 (57.4)	
Tertiary (over 13 years)	746 (38.7)	329 (36.8)	417 (40.3)	
*No. of skin diseases* ¤				<0.001
None	649 (33.6)	218 (24.4)	431 (41.6)	
1	688 (35.7)	328 (36.7)	360 (34.8)	
2	372 (19.3)	203 (22.7)	169 (16.3)	
3	151 (7.8)	96 (10.7)	55 (5.3)	
4 or more	70 (3.6)	49 (5.5)	21 (2.0)	
*Evaluation of need of treatment*				<0.001
No need of care	772 (40.0)	270 (30.3)	502 (48.5)	
Need a self-treatment	646 (33.5)	347 (38.9)	299 (28.9)	
Need care by a general practioner	472 (24.5)	256 (28.7)	216 (20.8)	
Need care in a special health care	38 (2.0)	19 (2.1)	19 (1.8)	

^*^Pearson's Chi-Squared test or Fisher's Exact test.

# Basic education (comprehensive schools), secondary level (upper secondary schools, vocational schools), tertiary level (universities, polytechnics).

¤ Skin findings which did not require treatment are excluded (for example benign skin tumors, androgenetic alopecia and erytematoteleangiectatic rosacea).

### Statistical analysis

The overall prevalence of all common skin diseases as well as the prevalence of each dermatologic diagnosis was calculated. The Chi-Square test, and Fisher's exact test when appropriate, was used to test difference in categorical variables. The modified Poisson regression model with a robust error variance was used to estimate crude and adjusted prevalence ratios and their 95% confidence intervals [15]. The adjusted model included both sex and educational level variables. Interactions for sex and the educational levels were also tested. All statistical analyses were performed with SAS 9.3 for Windows (The SAS Institute, Cary, NC, USA). All significance tests were two-tailed, and values of P<0.05 were considered statistically significant.

## Results

### Characteristics of the study population

A slight majority of the cohort members were females (53.7%, N = 1,036). Of them, 77% were married or cohabitant. The corresponding figure for males was 80%. Almost 60% of the study population had secondary level education, 38.7% had completed tertiary level and 2.8% had basic education (Table 1).

### Prevalence of skin findings and diseases

A whole-body skin examination revealed that every cohort member presented at least one pigmented naevus. Thus, benign skin tumors were the most common skin findings. About 12% of the cases showed multiple (>50) pigmented nevi. All other benign skin tumors are presented in Table 2. Androgenetic alopecia was frequent; it was found in 69% of the males, 27% of the cases being severe, 32% moderate and 38% mild.

**Table 2:** Prevalences of found skin diseases and skin findings in the study population, their total numbers and distribution by sex.

	Total	Male	Female	
	% (n)	% (n)	% (n)	*P*-value*
*Benign skin tumors* #	1930 (100)	894 (100)	1036 (100)	
Pigmented naevus, >50 nevi	222 (11.5)	116 (13.0)	106 (10.2)	0.060
Granuloma pyogenicum	0 (0.0)	0 (0.0)	0 (0.0)	
Blue naevus	26 (1.3)	8 (0.9)	18 (1.7)	0.109
Naevus spilus	90 (4.7)	39 (4.4)	51 (4.9)	0.561
Halo naevus	16 (0.8)	7 (0.8)	9 (0.9)	0.836
Dermatofibroma	428 (22.2)	155 (17.3)	273 (26.4)	<0.001
Cherry angioma	1158 (60.0)	476 (53.2)	682 (65.9)	<0.001
Keloid	52 (2.7)	24 (2.7)	28 (2.7)	0.980
Seborrheic keratosis	855 (44.3)	366 (40.9)	489 (47.2)	0.006
Lentigo senilis	262 (13.6)	90 (10.1)	172 (16.6)	<0.001
Lipoma	61 (3.2)	41 (4.6)	20 (1.9)	<0.001
Congenital melanocytic naevus	78 (4.0)	44 (4.9)	34 (3.3)	0.068
Naevus flammeus	495 (25.6)	195 (21.8)	300 (29.0)	<0.001
Cafe au lait spot	240 (12.4)	107 (12.0)	133 (12.8)	0.564
*Skin infections*	840 (43.5)	483 (54.0)	357 (34.5)	<0.001
Pityriasis versicolor	38 (2.0)	19 (2.1)	19 (1.8)	0.646
Onychomycosis	181 (9.4)	126 (14.1)	55 (5.3)	<0.001
Tinea pedis	517 (26.8)	327 (36.6)	190 (18.3)	<0.001
Tinea corporis	8 (0.4)	4 (0.4)	4 (0.4)	0.834
Folliculitis	116 (6.0)	86 (9.6)	30 (2.9)	<0.001
Pyoderma	1 (0.1)	0 (0.0)	1 (0.1)	0.353
Verruca plantaris	179 (9.3)	76 (8.5)	103 (9.9)	0.277
Verruca palmaris	51 (2.6)	22 (2.5)	29 (2.8)	0.644
*Sebaceus gland diseases*	515 (26.7)	210 (23.5)	305 (29.4)	0.003
Acne vulgaris	152 (7.9)	66 (7.4)	86 (8.3)	0.451
Comedo acne	26 (1.3)	8 (0.9)	18 (1.7)	0.109
Papulopustular acne	130 (6.7)	64 (7.2)	66 (6.4)	0.495
Cystic acne	8 (0.4)	4 (0.4)	4 (0.4)	0.836
Acne scars	188 (9.8)	103 (11.5)	85 (8.2)	0.015
Rosacea	292 (15.1)	94 (10.5)	198 (19.1)	<0.001
Erytematoteleangiectatic	242 (12.6)	78 (8.7)	164 (15.9)	<0.001
Papulopustular	45 (2.3)	12 (1.3)	33 (3.2)	0.007
Phymatotic	3 (0.2)	3 (0.3)	0 (0.0)	0.062
Ocular	1 (0.1)	0 (0.0)	1 (0.1)	0.352
Perioraldermatitis	14 (0.7)	1 (0.1)	13 (1.3)	0.003
*Eczemas and psoriasis*	563 (29.2)	295 (33.0)	268 (25.9)	<0.001
Eczemas	528 (27.4)	277 (31.0)	251 (24.2)	<0.001
Atopic eczema	93 (4.8)	35 (3.9)	58 (5.6)	0.084
Hand eczema	171 (8.9)	66 (7.4)	105 (10.1)	0.033
Seborrheic eczema	141 (7.3)	108 (12.1)	33 (3.2)	<0.001
Eczema nummulare	37 (1.9)	25 (2.8)	12 (1.2)	0.009
Eczema infectiosum	28 (1.5)	21 (2.3)	7 (0.7)	0.002
Contact eczema	19 (1.0)	2 (0.2)	17 (1.6)	0.002
Eczema staticum	15 (0.8)	8 (0.9)	7 (0.7)	0.586
Neurodermatitis	35 (1.8)	26 (2.9)	9 (0.9)	<0.001
Psoriasis	40 (2.1)	23 (2.6)	17 (1.6)	0.202
*Hair follicle diseases*	654 (33.9)	629 (70.4)	25 (2.4)	a)<0.001
Alopecia areata	7 (0.4)	5 (0.6)	2 (0.2)	0.182
Androgenetic alopecia	627 (32.5)	613 (68.6)	14 (1.4)	<0.001
Hyperhidrosis	42 (2.2)	32 (3.6)	10 (1.0)	<0.001
*Autoimmune diseases*	81 (4.2)	32 (3.6)	49 (4.7)	0.209
Lichen ruber planus	13 (0.7)	8 (0.9)	5 (0.5)	0.270
Dermatitis herpetiformis	6 (0.3)	2 (0.2)	4 (0.4)	0.523
DLE	1 (0.1)	0 (0.0)	1 (0.1)	0.353
SLE	1 (0.1)	0 (0.0)	1 (0.1)	0.353
Vitiligo	32 (1.7)	10 (1.1)	22 (2.1)	0.085
Urticaria	10 (0.5)	5 (0.6)	5 (0.5)	0.815
*Malign skin tumors*	12 (0.6)	7 (0.8)	5 (0.5)	0.403
Melanoma	1 (0.1)	1 (0.1)	0.0 (0)	0.353
Solar keratosis	12 (0.6)	7 (0.8)	5 (0.5)	0.403
Basal cell carcinoma	7 (0.4)	2 (0.2)	5 (0.5)	0.346

^*^P-value for difference of prevalence between males and females evaluated using Pearson's Chi-Squared test or Fisher's Exact test.

# All study cases had at least one pigmented naevus.

The most common skin disease was tinea pedis, found in 26.8% of all cohort members; 20% of them had also onychomycosis. Total prevalence of onychomycosis was 9.4%. Rosacea was also quite common, affecting 15.1%, while acne vulgaris was found in 7.9% of the cohort members.

The most commonly seen eczemas were hand eczema (8.9%) and seborrhoeic eczema (7.3%). The prevalence of atopic eczema was 4.8%, the mean EASI value being 4.7 (range 0.1–36), reflecting mild diseases. The prevalence of psoriasis was 2.1%, mean PASI being 3.1 (range 0.1–13.7). Approximately one in every three of all psoriatics reported joint symptoms (33.3%), and a third (30.6%) had nail deformities. The most usual autoimmune skin disease was vitiligo, found in 1.6% of study cases.

Already at the age of 46 years there were twelve (0.62%) cases of solar keratosis in the study population. Finally, seven (0.36%) basal cell carcinomas and one malignant melanoma (0.05%) were also found (Table 2).

Furthermore, there were several other skin findings that were rare or transient in nature, and because of that they have not been included in Table 2. These skin disorders were for example herpes simplex, pustulosis palmoplantaris, acanthosis nigricans and polymorphic light eruption.

Finally, we analyzed if some study participants had multiple skin diseases; this revealed that 39% of males and 24% of females had more than one diagnosed skin disease (Table 1).

### Distribution of skin findings and diseases by sex

When compared to females, seborrhoeic eczema (RR 3.8), eczema nummulare (RR 2.4), eczema infectiosum (RR 3.4) and neurodermatitis (RR 3.4) were more common in males. Also the two most common mycoses, tinea pedis (RR 2.0) and onychomycosis (RR 2.6) predominated in males. Similarly, folliculitis (RR 3.3) and hyperhidrosis affected more males (RR 3.7) than females. In contrast, contact eczema (RR 0.1) and hand eczema (RR 0.7) were less common among males. Certain benign skin tumors, such as dermatofibromas (RR 0.7), cherry angiomas (RR 0.8), seborrheic keratosis (RR 0.9), lentigo senilis (RR 0.6) and naevus flammeus (RR 0.8), were less common among males than females. Lipoma was more common in males (RR 2.4), while rosacea predominated in females (RR 1.9). No significant difference in sex distribution was found in atopic dermatitis and psoriasis (Table 3).

**Table 3:** The effect of male sex to the prevalence of skin findings and diseases, compared to females.

	Unadjusted RRs		Adjusted* RRs	
	RR	95% CI	aRR	95% CI
*Benign skin tumor*				
Dermatofibroma	0.66	0.55–0.78	0.66	0.55–0.79
Cherry angioma	0.81	0.75–0.87	0.81	0.75–0.88
Seborrheic keratosis	0.87	0.78–0.96	0.87	0.78–0.96
Lentigo senilis	0.61	0.48–0.77	0.61	0.48–0.77
Lipoma	2.38	1.40–4.02	2.40	1.42–4.06
Naevus flammeus	0.75	0.64–0.88	0.75	0.64–0.88
*Skin infections*	1.57	1.41–1.74	1.56	1.40–1.73
Onychomycosis	2.65	1.96–3.60	2.63	1.94–3.56
Tinea pedis	1.99	1.71–2.33	1.98	1.70–2.31
Folliculitis	3.32	2.21–4.98	3.27	2.18–4.91
*Sebaceus gland diseases*	0.80	0.69–0.93	0.79	0.68–0.92
Acne scars	1.40	1.07–1.84	1.39	1.06–1.83
Rosacea	0.55	0.44–0.69	0.54	0.43–0.68
Erytematoteleangiectatic	0.55	0.43–0.71	0.54	0.42 – 0.70
Papulopustular	0.42	0.22–0.81	0.42	0.22–0.81
Perioraldermatitis	0.09	0.01–0.68	0.09	0.01–0.70
*Eczemas*	1.28	1.11–1.48	1.28	1.10–1.48
Atopic eczema	0.70	0.46–1.05	0.70	0.47–1.06
Hand eczema	0.73	0.54–0.98	0.73	0.54–0.98
Seborrheic eczema	3.79	2.59–5.54	3.78	2.59–5.53
Eczema nummulare	2.41	1.22–4.77	2.37	1.21–4.67
Eczema infectiosum	3.47	1.48–8.13	3.36	1.43–7.86
Contact eczema	0.14	0.03–0.59	0.13	0.03–0.58
Neurodermatitis	3.34	1.58–7.10	3.36	1.59–7.10
*Hair follicle diseases*	29.16	19.75–43.04	29.13	19.73–43.02
Androgenetic alopecia	50.74	30.10–85.53	50.73	30.09–85.52
Hyperhidrosis	3.71	1.83–7.50	3.68	1.82–7.47

Abbrevations: RR, relative risk; aRR, adjusted relative risk; CI, confidence interval.

* Adjusted for education.

### Socioeconomic status and the prevalence of skin diseases

We analyzed the association of socioeconomic status, defined as education level, with the prevalence of skin diseases. When compared with tertiary education level, tinea pedis (RR 1.28), onychomycosis (RR 1.34), eczema infectiosum (RR 3.5) and folliculitis (RR 1.63) were more common among those with secondary level education (P<0.05). In comparison between tertiary and basic education, the corresponding values were: tinea pedis (RR 1.63), onychomycosis (RR 1.94) and eczema infectiosum (RR 10.3) (P<0.05).

### The need of treatment

Skin diseases defined as serious enough in order to be in need of care was high being 59.9% (N = 1156). Importantly, almost half (40.8%, N = 472) were referred to a general practitioner or occupational health care, and 3.3% (N = 38) to secondary care. However, about half of the cases in need of treatment were mild and were given advice on self-care treatment (N = 646). The need for treatment was higher among males (70%) than females (52%) (P<0.001) as well as in lower educational class (basic education 61%, secondary level 63% and tertiary level 56%) (P<0.05) (Table 1).

## Discussion

The novel and most important finding of our epidemiological study among nearly 2,000 subjects (aged 45–46 years) of NFBC 1966 was the high prevalence of doctor-diagnosed skin diseases (60%) serious enough to need further treatment, either by a physician or self-treatment. Thus, skin disorders are unquestionably one of the most common reasons of morbidity in an adult general population. In addition, we found that socioeconomic status and sex are associated with the prevalence of some skin diseases.

In this study, the need for treatment was higher compared to previous British and German population based studies: the prevalence of skin diseases requiring any therapy in these earlier reports was 23% and 27%, respectively [1], [6]. However, in Rea and co-worker's study the population was relatively small (N = 614), part of the skin findings were diagnosed by a nurse, and some skin findings were classified as trivial, not justifying medical attention. The recent study by Augustin and co-workers (2011) included exclusively health ensured employed cases with a wide age distribution (16 years to 70 years). In contrast, a clear advantage of our study was the pure population-based study design with altogether 2,000 unselected members who were all examined by an expert. We determined “skin disease” as a condition that required follow-up or treatment, and thus for example benign tumors, androgenetic alopecia and erytematoteleangiectatic rosacea were excluded from this category. Nevertheless, we decided to recognize and report all cutaneous findings since they may have clinical importance. For example, tinea pedis may predispose to severe infections or present a primary sign of general diseases such as diabetes, but patients do not seek medical care due to the triviality of the symptoms. On the other hand, pigmented nevi, seborrheic keratosis and other benign tumors cause numerous visits to health care. In general, doctor-seeking behavior is determined not only by the severity of condition, but is also influenced by personal, financial and cultural factors.

The other remarkable finding in our study was that the need of treatment was significantly higher with low socioeconomic status. Interestingly, we found that skin infections and specific eczemas were more common in lower socioeconomic status compared to higher socioeconomic status. This is in line with the general postulate of Adler and others: the lower the socioeconomic status, the higher the risk of diseases [16]. However, the relationship between socioeconomic status and skin diseases has remained controversial: Dalstra and co-workers did not find any association between socioeconomic status and skin diseases [17]; on the other hand, the prevalences of atopic disease and skin cancer have been reported to be higher in high socioeconomic status [17], [18], [19], [20].

The majority of epidemiological studies in dermatology have focused on the prevalence of single skin diseases, the most commonly investigated ones being atopic eczema, psoriasis, acne vulgaris, rosacea, and hand eczema [21], [22], [23], [24], [25], [26], [27]. It is noteworthy that our study is the first one in Scandinavia covering widely the prevalence of cutaneous findings. In our study, psoriasis was seen in two percent of the cases, which is in line with previous studies [6], [28]. Therefore, our finding supports the existing understanding that psoriasis is not dependent on a single characteristic of any geographical region [5]. Hand eczema was seen in 8.9% in our cohort, more often in females, in line with previous reports [29], [30], [31]. In contrast, atopic eczema was clearly more common (4.8%) than previously reported (from 1.3% to 2.9%) [6], [32]. The difference is understandable because the incidence of atopic eczema varies between countries and continents [21]. Interestingly, as many as one in every six (15%) of our cohort members presented rosacea. Existing epidemiological data on rosacea is controversial, with reported prevalences varying between 2% and 22% [6], [26], [33]. An increased risk for rosacea has been connected more to female sex [10], fair-skinned population [34] and middle-aged people. The last two of these describe our cohort members and could therefore explain our high prevalence.

Our current work brings certain new considerations to the existing epidemiological literature with regard to more sparsely studied dermatological entities. In our study, over two thirds (69%) of males had some form of hair loss while nearly one third (27%, N = 171) had severe androgenetic alopecia. In comparison, in an Italian study (N = 1390), the prevalence of androgenetic alopecia consisting of frontal and vertex androgenetic alopecia (severe) was only 19% (men under 55 years, N = 52) [35]. Early androgenetic alopecia can work as a marker of insulin resistance [36]. Androgenetic alopecia has also been shown to be associated with several other diseases [37] and to decrease the quality of life [38].

An unexpected high number of middle-aged cohort members (44.3%) showed seborrheic keratosis. Albeit benign in nature, its presence may cause distress to patients due to fear of malignant tumors; even today, it is often surgically removed in vain. Multiple pigmented nevi (>50) were found in up to 11.5% of the cohort members, supporting the previous results [39], [40]. The total number of nevi has been shown to be one of the strongest risk factors of melanoma [41], and the incidence of malignant melanoma has increased noticeably in recent decades [42]. Melanoma has been reported to be more common in Scandinavian countries and to have female predominance [43].

In our study, tinea pedis (26%) was more frequent than previously reported (5–15%) in other industrial countries [6], [44]. This is an important finding because it is associated with other diseases, e.g. erysipelas [45], and its treatment among diabetics is important. Tinea pedis influences people widely, being also common in athletics [46]. Our high prevalence may be explained by the fact that also minor manifestations, still fulfilling the clinical criterion of tinea pedis, were included.

The predominance of mycotic and bacterial skin infections as well as almost all eczemas in male cohort members, and specific benign skin tumors such as dermatofibromas and seborrheic keratosis in female cohort members, is in line with the findings with a German study [6]. In contrast to the study by Augustin and co-workers (2011) we found that hand eczema and rosacea affected females more often than males. Our important finding was that the overall need of treatment was much higher among males (70%) than females (52%).

The major strength of this study was the coverage of a wide general population with a relatively high (61%) participation rate, which means that our results are generalizable. We have good statistical power thanks to the large sample size. As a major difference to many previous epidemiological studies [6], [47], [48], our current work was not limited to any population subgroups (e.g. employed, children, mental health or patients with psoriasis). Another special advantage was the accurate clinical whole-body skin evaluation performed for every case by an experienced resident or specialist in dermatology instead of the self-reporting questionnaires used in some previous studies [49], [50], [51]. Finally, we were able to analyze the association of skin diseases with socioeconomic status using the level of education, which is reported to be most reliable indicator of socioeconomic status [52]. We concede as a weakness of our study that all those invited did not participate in the clinical examination. All the diagnoses were based solely on the clinical picture since medical history, histologic analysis and microbiologic culture or microscope analysis were not available due to the study setting. Furthermore, the investigators had to reach the diagnosis for the study purpose during one out-hospital visit without possibilities for therapeutical tests or repetitive visits, despite their importance in dermatology.

In conclusion, our major finding was a high prevalence of skin diseases requiring treatment. In clinical practice this means that careful skin evaluation is an important and essential part of the medical examination. Due to comprehensive birth cohort study design and a satisfactory, high participation rate (61%), we underline that our valuable results are valid and generalizable. We found that low socioeconomic status was a risk factor for certain skin diseases. The surprisingly high overall prevalence of skin diseases and the great need of treatment warrant further epidemiologic studies on the factors which are associated with skin diseases.
